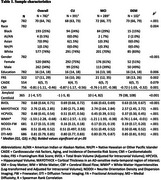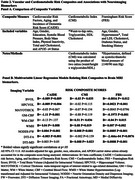# Evaluating associations between vascular and cardiometabolic‐based risk composites with aging and Alzheimer's disease related neuroimaging biomarkers in a heterogeneous community‐based cohort

**DOI:** 10.1002/alz70856_106599

**Published:** 2026-01-08

**Authors:** Kathryn H Alphin, Melissa M. Rundle, Chinedu T Udeh‐Momoh, Da Ma, Timothy M. Hughes, Trey R. Bateman, Suzanne Craft, Marc D. Rudolph

**Affiliations:** ^1^ Wake Forest University School of Medicine, Winston‐Salem, NC, USA

## Abstract

**Background:**

Research examining how vascular and cardiometabolic‐based risk composites like the Cardiovascular risk factors, Aging, and Incidence of Dementia Risk Score (CAIDE), Cardiometabolic Index (CMI), and Framingham Risk Score (FRS) are associated with AD‐related neuroimaging biomarkers in diverse cohorts is limited. We thus compared CAIDE, CMI, and FRS composites to neuroimaging‐based neurodegenerative markers in a heterogeneous community‐based cohort.

**Methods:**

Adults 55 and older were recruited into the Clinical Core of the Wake Forest Alzheimer's Disease Research Center (WFADRC) between 2016 and 2024 and underwent standard evaluation in accordance with the National Alzheimer's Coordinating Center (NACC) protocols (Table 1). Demographics, vital measurements, and lifestyle information were collected to calculate CAIDE, CMI, and FRS. Baseline neuroimaging measures of total brain and hippocampal volume (BVOL; HPCVOL), AD‐specific meta‐temporal lobe cortical thickness, free water from neurite orientation dispersion and density imaging (NODDI‐FW), and cerebral blood flow in gray and white matter (GMCBF; WMCBF) were ascertained. Baseline measures of amyloid PET deposition were also examined. General linear models examined if CAIDE, CMI, and FRS risk composites (1) differed by sex, self‐reported race/ethnicity, or *APOE*‐ ε4 status, and (2) were associated with baseline neuroimaging metrics. Models were adjusted for age, gender, race, education, and *APOE*‐ ε4 carrier status when not already captured by a composite score.

**Results:**

All risk scores positively correlated with each other (*p* <0.001). CAIDE was higher in males and Black participants, CMI was higher among females and Black participants (*p* <.05), while FRS was higher in males (*p<.001*) independent of gender. All risk scores were negatively associated with BVOL (*p* <.05; Table 2) and positively correlated with WMH (*p* <.05) and NODDI‐FW (*p* <.001). CAIDE and FRS were negatively correlated with hippocampal volume (*p* <.05), GM‐CBF (*p* <.001), and DTI‐FA (*p* <.05). CMI and FRS were positively correlated with WM‐CBF (*p* <.05) and DTI‐MD (*p* <.05). CMI was positively correlated with WM‐CBF (*p* <.05), while FRS showed a negative relationship (*p* <.05). Risk scores were not associated with measures of cortical thickness or amyloid deposition.

**Conclusions:**

Risk scores assessed were broadly associated with poorer overall brain health, particularly measures capturing vascular pathology. Overall, the neuroimaging measures were most strongly associated with the FRS.